# Value of histopathologic analysis of subcutis excisions by general practitioners

**DOI:** 10.1186/1471-2296-8-5

**Published:** 2007-01-26

**Authors:** Pieter AJ Buis, Wim Verweij, Paul J van Diest

**Affiliations:** 1 General Practice H. de Manstraat, Harderwijk, The Netherlands; 2 SALTRO, Utrecht, The Netherlands; 3 Department of Pathology, VU University Medical Center, Amsterdam, The Netherlands; 4 Department of Pathology, University Medical Center, Utrecht, The Netherlands

## Abstract

**Background:**

Only around 60% of skin lesions excised by GPs are referred to a pathologist. Clinical diagnoses of skin excisions by GPs may not be very accurate. Subcutis excisions are rarely done by GPs, and there is hence little information in the literature on the histopathological yield of subcutis excisions by GPs with regard to malignancies.

The aim of this study was to evaluate the yield of histopathological investigation of a relatively large group of subcutis excisions by GPs, with special emphasis on discrepancies between clinical and histopathological diagnoses of malignancy.

**Methods:**

We investigated a series of 90 subcutis excisions, which was derived from a database of consecutive GP submissions from the years 1999–2000 where in the same time period 4595 skin excisions were performed by the same group of GPs. This underlines the apparent reluctance of GPs to perform subcutis excisions.

**Results:**

The final diagnosis was benign in 88 cases (97.8%) and malignant in 2 cases (2.2%). Seven cases had no clinical diagnosis, all of which were benign. Of the 83 clinically benign cases, 81 (97.6%) were indeed benign and 2 (2.4%) were malignant: one Merkel cell carcinoma and one dermatofibrosarcoma protuberans. The former was clinically thought to be a lipoma, and the latter a trichilemmal cyst. The dermatofibrosarcoma protuberans presented at the age of 27, and the Merkel cell carcinoma at the age of 60. Both were incompletely removed and required re-excision by a surgical oncologist.

**Conclusion:**

Histopathological investigation of subcutis excisions by GPs yields unexpected and rare malignancies in about 2% of cases that may initially be excised inadequately. Based on these data, and because of the relatively rareness of these type of excisions, it could be argued that it may be worthwhile to have all subcutis excisions by GPs routinely investigated by histopathology.

## Background

Most general practitioners (GPs) do not submit all their excisions for histopathological investigation, apparently relying on their clinical assessment of the benign nature of some lesions. It has been estimated that only around 60% of lesions excised by GPs are referred to a pathologist [1,2]. Studies have shown that clinical diagnoses of skin excisions by GPs may not be very accurate. Some studies found a discrepancy rate of at least 30% between clinical and histopathological diagnoses by GPs on skin excisions [3,4]. We showed in a previous study that skin excisions by GPs harboured about 5% of often unexpected (pre)malignancies, and argued that all skin excisions must therefore be routinely investigated by histopathology in order not to miss serious malignancies [5].

Subcutis excisions are rarely done by GPs, and there is hence little information in the literature on the histopathological yield of subcutis excisions by GPs with regard to malignancies (all probably unexpected), let alone its cost-effectiveness. However, also primary incomplete excision of a malignancy in a subcutis excision could lead to untreatable local or metastasised recurrences, and some malignancies require additional treatment besides local excision such as Merkel cell carcinoma (sentinel lymph node procedure [6]) or chemotherapy (lymphomas).

The only way to have a primary diagnosis of malignancy, and to know whether additional treatment is required, is to investigate all subcutis excisions. So, should all subcutis excisions by GPs indeed be histopathologically investigated? For skin excisions, it has been argued that this may not be worth the large increase in workload and costs [7], but since subcutis excisions are much less frequently done by GPs, this argument may be less valid for subcutis excisions.

The aim of this study was therefore to evaluate the yield of histopathological investigation of a relatively large group of subcutis excisions by GPs, with special emphasis on discrepancies between clinical and histopathological diagnoses of malignancy.

## Methods

The SALTRO is a general practice laboratory for clinical chemistry, pathology and haematology in Utrecht, The Netherlands, serving many GPs in the greater Utrecht region. GPs performing minor surgery submit most of the resected specimens to the SALTRO for histopathological investigation, which is performed at the Department of Pathology of the VU University Medical Center in Amsterdam, The Netherlands. From the years 1999 and 2000, all pathology reports from histological submissions by GPs to the SALTRO were reviewed. Multiple submissions under the same entry number were split up so that each resection or biopsy could be analysed separately. This resulted in 4595 skin excisions (from which the results have been reported before [5]) and 90 excisions containing no skin but only subcutaneous tissue. For each of these consecutive "subcutis" entries, the clinical diagnosis was noted and grouped as benign, malignant, or unknown. All final histopathological diagnoses were noted as well and grouped as benign or malignant. The clinical diagnosis status (benign, malignant, unknown) was compared with the final diagnosis status. Further, the detailed clinical diagnosis was compared with the final detailed histopathological diagnosis.

## Results

As shown in table 1, the most frequent clinical diagnosis was lipoma (n = 51, 56.7%), followed by trichilemmal cyst (n = 24, 26.7%). For seven cases (7.8%), no clinical diagnosis was provided, and no case was suspected to be malignant.

**Table 1:** Clinical diagnosis of 90 subcutis excisions by general practitioners

	**Frequency (%)**	**Confirmed by histopathology (%)**
Unknown	7 (7.8%)	
Trichilemmal cyst	24 (26.7%)	10 (42%)
Epidermal cyst	1 (1.1%)	0 (0%)
Cyst	2 (2.2%)	0 (0%)
Fibroma	2 (2.2%)	0 (0%)
Lipoma	51 (56.7%)	41 (80%)
Scar	1 (1.1%)	0 (0%)
Pilomatricoma	2 (2.2%)	2 (100%)

Total	90	

The final histopathological diagnosis was benign in 88 cases (97.8%). The most frequent benign diagnoses (table 2) were lipoma (n = 47, 52.2%), trichilemmal cyst (n = 12, 13.3%), and epidermal cyst (n = 9, 10%), and leiomyoma (n = 4, 4.4%).

**Table 2:** Final histological diagnosis of 90 subcutis excisions by general practitioners

	**Frequency**	**Percentage**
*Benign*		
Dermatofibroma	1	1.1
Digital mucinous cyst	1	1.1
Epidermal cyst	9	10
Median raphe cyst	1	1.1
Neurofibroma	2	2.2
Trichilemmal cyst	12	13.3
Ganglion	1	1.1
Hemangioma	1	1.1
Hydrocystoma	1	1.1
Leiomyoma	4	4.4
Lipoma	47	52.2
Lymph node	1	1.1
Panniculitis	2	2.2
Pilomatricoma	2	2.2
Giant cell tumor	2	2.2
Schwannoma	1	1.1
		
*Malignant*		
Dermatofibrosarcoma protuberans	1	1.1
Merkel cell carcinoma	1	1.1

Total	90	100.0

Two cases (2.2%) were malignant, one Merkel cell carcinoma and one dermatofibrosarcoma protuberans. Both these lesions were incompletely removed and required re-excision by a surgical oncologist. The former was clinically thought to be lipoma, and the latter a trichilemmal cyst. The dermatofibrosarcoma protuberans presented at the age of 27, and the Merkel cell carcinoma at the age of 60. So, of the 83 clinically benign cases, 81 (97.6%) were indeed benign and 2 (2.4%) were malignant. The seven cases without clinical diagnosis were all benign.

The positive predictive value of the clinical diagnoses grouped as benign/malignant was 0% as no lesion was clinically suspected to be malignant, and the negative predictive value was 97.2%. The detailed clinical diagnosis matched with the exact histopathological diagnosis in 60 of the 90 cases, leading to an overall accuracy of the detailed clinical diagnosis of 67%.

## Discussion

Routine histopathological investigation of excisions by GPs is controversial. It is well known that most GPs do not submit all excisions for histopathological investigation, apparently relying on their clinical assessment of the benign nature of some lesions. Some studies reported that up to 40% of lesions excised by GPs are not referred to a pathologist [1,2,8]. Several studies have focussed on the yield of histopathological investigation of skin excisions by GPs [3-5], some arguing that all skin excisions should be referred for histopathology in order not to miss serious malignancies [5]. Few data on subcutis excisions are available, probably at least in part due to the fact that these are rarely done by GPs. The aim of this study was to therefore evaluate the yield of histopathological investigation in a relatively large set of subcutis excisions by GPs.

We investigated a series of 90 subcutis excisions, which was derived from a database where in the same time period 4595 skin excisions were performed by the same group of GPs. This underlines the apparent reluctance of GPs to perform subcutis excisions. The most frequent clinical diagnoses were lipoma and trichilemmal cyst. No cases were suspected to be malignant, which is well understandable, as such cases would as a rule be referred. In 2.2% of excisions, the final histopathological diagnosis was malignant. Both these were unexpected, and concerned rare malignancies for which the excision with subsequent histopathology were clinically quite relevant. For the Merkel cell carcinoma, a sentinel node would have been indicated [6]. Both malignancies were incompletely removed and required re-excision by a surgical oncologist. Not diagnosing these malignancies by histopathology would later most likely have resulted in serious problems.

The fact that both malignancies were unexpected (positive predictive value 0%) indicates that the clinical assessment of subcutis lesions by GPs is not 100% reliable as previously shown for skin excisions [5]. This finding is not unique for GPs, as even dermatologists face the same problem for skin excisions [3,4], and dermatologists and surgeons may well have similar problems with subcutis excisions.

On a more detailed level, 60/90 of the clinical diagnoses were confirmed by histopathology (overall accuracy 67%). The accuracy of the clinically most frequent diagnosis lipoma was 80% (41/51 cases confirmed by histopathology), and of the clinically second most frequent diagnosis trichilemmal cyst 42% (10/24 confirmed by histopathology). Interestingly, both cases that were clinically diagnosed as pilomatricoma were indeed as such diagnoses by histopathology.

In our previous study [5], we showed that age can help to select those patients at highest risk for unexpected malignancies (>40). For subcutis excisions, this cannot be concluded. One case presented at the age of 27, and the other at the age of 60. However, in view of these low numbers, we have to be careful here.

The question therefore arises whether all subcutis excisions need to be submitted for histopathological evaluation. This would obviously ensure detection of all malignancies, and prevent untreatable recurrences. Naturally, this involves costs, but this may be neglected since the number of subcutis excisions by GPs is quite low in comparison with skin excisions. Overall, there seem to be many arguments to submit all excised subcutis material for histopathological investigation.

One drawback to this study is that we are not aware of the submission attitude of the GPs involved in this study for subcutis excisions, but we speculate that the percentage of submissions for histopathology for subcutis excisions is higher than that for skin excisions.

## Conclusion

Histopathological investigation of subcutis excisions by GPs yields about 2% of serious and unexpected malignancies. This indicates that clinical assessment of subcutis lesions by GPs is insufficiently reliable to allow some subcutis excisions to be kept from histopathological investigation, and that all subcutis excisions by GPs deserve to be routinely investigated by histopathology in order not to miss serious malignancies.

## Abbreviations

SALTRO: Stichting Artsen Laboratorium en Trombosedienst (a general practice laboratory for clinical chemistry, pathology and haematology)

VU: Vrije Universiteit (Free University, Amsterdam)

GP: general practitioner

## Competing interests

The author(s) declare that they have no competing interests.

## Authors' contributions

PAJB designed the study, did data analysis, and drafted the menuscript

WV performed data acquisition and participated in the writing

PJvD conceived of the study, participated in its design and coordination, helped in data analysis, and participated in the writing.

All authors read and approved the final manuscript.

## Pre-publication history

The pre-publication history for this paper can be accessed here:


